# Pituitary adenylate cyclase-activating polypeptide (PACAP) contributes to the proliferation of hematopoietic progenitor cells in murine bone marrow via PACAP-specific receptor

**DOI:** 10.1038/srep22373

**Published:** 2016-02-29

**Authors:** Zhifang Xu, Hirokazu Ohtaki, Jun Watanabe, Kazuyuki Miyamoto, Norimitsu Murai, Shun Sasaki, Minako Matsumoto, Hitoshi Hashimoto, Yutaka Hiraizumi, Satoshi Numazawa, Seiji Shioda

**Affiliations:** 1 Department of Anatomy, Showa University School of Medicine, 1-5-8 Hatanodai, Shinagawa, Tokyo 142-8555, Japan; 2 Division of Toxicology, Department of Pharmacology, Toxicology and Therapeutics, Showa University School of Pharmacy, 1-5-8 Hatanodai, Shinagawa, Tokyo 142-8555, Japan; 3 Center for Biotechnology, Showa University, 1-5-8 Hatanodai, Shinagawa, Tokyo 142-8555, Japan; 4 Laboratory of Molecular Neuropharmacology, Graduate School of Pharmaceutical Sciences, Osaka University, 1-6 Yamadaoka, Suita, Osaka 565-0871, Japan; 5 Department of Orthopaedic Surgery, Showa University School of Medicine, 1-5-8 Hatanodai, Shinagawa, Tokyo 142-8555, Japan; 6 Peptide Drug Innovation, Global Research Center for Innovative Life Science, Hoshi University School of Pharmacy and Pharmaceutical Sciences, 2-4-41 Ebara, Shinagawa, Tokyo 142-8501, Japan; 7 Division of Cellular Signaling, Institute for Advanced Medical Research, Keio University School of Medicine, 35 Shinanomachi, Shinjuku, Tokyo 160-8582, Japan

## Abstract

Pituitary adenylate cyclase-activating polypeptide (PACAP, encoded by *adcyap1*) plays an important role in ectodermal development. However, the involvement of PACAP in the development of other germ layers is still unclear. This study assessed the expression of a PACAP-specific receptor (PAC1) gene and protein in mouse bone marrow (BM). Cells strongly expressing PAC1^+^ were large in size, had oval nuclei, and merged with CD34^+^ cells, suggesting that the former were hematopoietic progenitor cells (HPCs). Compared with wild-type mice, *adcyap1*^*−/−*^ mice exhibited lower multiple potential progenitor cell populations and cell frequency in the S-phase of the cell cycle. Exogenous PACAP38 significantly increased the numbers of colony forming unit-granulocyte/macrophage progenitor cells (CFU-GM) with two peaks in semi-solid culture. PACAP also increased the expression of cyclinD1 and Ki67 mRNAs. These increases were completely and partially inhibited by the PACAP receptor antagonists, PACAP6-38 and VIP6-28, respectively. Little or no *adcyap1* was expressed in BM and the number of CFU-GM colonies was similar in *adcyap1*^*−/−*^ and wild-type mice. However, PACAP mRNA and protein were expressed in paravertebral sympathetic ganglia, which innervate tibial BM, and in the sympathetic fibers of BM cavity. These results suggested that sympathetic nerve innervation may be responsible for PACAP-regulated hematopoiesis in BM, mainly via PAC1.

Pituitary adenylate cyclase-activating polypeptide (PACAP) is a multifunctional neuropeptide belonging to the glucagon-secretin-vasoactive intestinal peptide (VIP) family. PACAP binds to three G-protein coupled receptors, a higher affinity PACAP-specific receptor (PAC1), and two VIP/PACAP receptors (VPAC1 and VPAC2), which display a 1,000-fold lower affinity to PACAP than PAC1^1^. PACAP has been shown to be involved in the suppression of the death of neural^2^,^3^,^4^,^5^,^6^,^7^,^8^ and other types of cells, the modulation or suppression of immune and inflammatory responses^9^,^10^,^11^,^12^,^13^, and the dilation of vessels and bronchi^14^,^15^, as well as in psychomotor control^16^,^17^.

PACAP is also known to play an important role in the development of cells of ectodermal lineage. The gene encoding PAC1 (*adcyap1r1*) is expressed in neuroepithelial cells of neural tubes, an area of cell proliferation, in embryonic day 9.5 mice^18^. Exogenous PACAP was shown to enhance the differentiation of neural progenitor cells (NPCs) into astrocytes *in vitro*^19^. Activation of PAC1 by PACAP inhibited cerebellar granule cell migration during postnatal maturation in rats^20^. Although nestin-positive NPCs of the subventricular zone (SVZ) were immunopositive for PAC1, PAC1 expression was transiently diminished in doublecortin-positive migrating neuroblasts of the rostral migratory stream^21^. Results showing that the dentin layer is thinner in infant *adcyap1* deficient (*adcyap1*^*−/−*^) than wild-type mice suggest that PACAP may contribute to odontoblast development^22^. Mouse embryonic stem (ES) cells were found to be positive for *adcyap1r1* and *vipr2*, which encodes VPAC2^23^, suggesting that PACAP may be involved in development of other germ cell layers. To date, however, one study has reported that PACAP over-expression inhibited megakaryopoiesis mediated by VPAC1^24^,^25^. We recently found that *adcyap1* and *adcyap1r1* were expressed in cultured human bone marrow mesenchymal stromal cells (hBMSCs) stimulated with interferon-γ, but not in untreated cells^26^. As hBMSCs occupy a hematopoietic niche^27^, these findings suggest that PACAP may be involved in bone marrow (BM) function.

BM is a predominant hematopoietic organ. Hematopoiesis in BM first occurs during the middle fetal period and continues throughout life. All hematopoietic cells originate from pluripotent hematopoietic stem cells (HSCs). HSCs comprise a small population in the BM and sit atop a hierarchy of hematopoietic progenitor cells (HPCs) that become progressively restricted to several or single lineage(s)^28^. The maturation of hematopoietic cells is controlled by niches consisting of cells and humoral and cellular membrane factors in the marrow stromal compartments^27^,^29^,^30^,^31^,^32^. However, the regulators of hematopoietic niches and factors have not been fully determined in detail.

This study assessed the presence of PAC1 expression in mouse BM. In particular, strong PAC1 immunopositivity was observed in larger size cells with oval nuclei that merged with CD34^+^ cells, suggesting that the former were HPCs. BM in a*dcyap1*^*−/−*^ mice exhibited lower multiple potential progenitor cell populations and cell frequency in the S-phase of the cell cycle compared with BM in wild-type mice. Exogenous PACAP38 significantly increased the numbers of colony forming unit-granulocyte/macrophage progenitor cells (CFU-GM) with two peaks mediated by PAC1 and VPAC2 in semi-solid culture. PACAP also increased the expression of cell-cycle related cyclin D1 (*ccnd1*) and Ki67 (*mki67*) genes. Taken together, these findings suggested that PACAP in BM may derive from innervating sympathetic fibers.

## Results

### PAC1 gene expression and localization in murine BM

**Figure 1:**
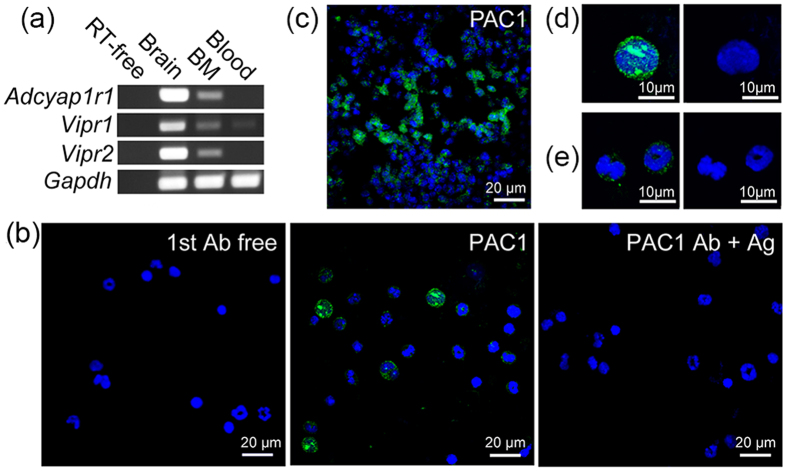
Expression of PACAP receptors in mouse BM. (**a**) Expression of genes encoding the three PACAP receptors (*adcyap1r1*, *vipr1* and *vipr2*) in BM and blood of mice. RT-free indicates no reverse transcriptase (negative control), with brain sample used as a positive control for receptor expression. Results were normalized relative to expression of *gapdh*. (**b**) Immunostaining of total BM smears with antibody to PAC1. PAC1-like immunoreactions (ir, *green*) were observed in the BM (*middle*, PAC1 Ab), but not in the absence of primary antibody (first Ab free) or when antibody was pre-absorbed with antigen (PAC1 Ab + Ag). (**c**) PAC1-ir (*green*) in decalcified BM frozen sections. (**d,e**) Cell types differ in intensity of PAC1-ir (*green*). PAC1^+^ cells with stronger intensity of PAC1 (*left* in **d**) were larger in size and had light oval nuclei (*right* in **d**), whereas those with weaker intensity (*left* in **e**) were smaller in size and had donut- and band-like nuclei (*right* in **e**). The blue color represents staining of nuclei with DAPI. Scale bars, 20 μm (**b,c**), 10 μm **(d,e**).

The initial step in investigating the association between PACAP and hematopoietic cells was to determine the expression of genes encoding the three PACAP receptors, *vipr1* (which encodes VPAC1), *vipr2* and *adcyap1r1*, in nucleated BM cells and blood (Fig. 1a). BM was positive for all three receptors, but to a lesser extent than brain (positive control), whereas blood samples were positive only for *vipr1*. Consistent with gene expression, PAC1 immunoreactivity was observed in nucleated cells of BM smears (Fig. 1b) and decalcified BM sections (Fig. 1c). The specificity of these reactions was demonstrated by omitting the primary antibody and by antigen pre-absorbance (Fig. 1b and Supplementary Fig. S1A,B). BM cells positive for PAC1 were observed around the sinusoidal lumen and formed cluster-like aggregates (Fig. 1b). PAC1 immunopositivity in smear sections exhibited different intensities, depending on cell size and nuclear shapes, with stronger intensity indicative of larger sized cells and light oval nuclei (Fig. 1d) and weaker intensity indicative of smaller sized cells and donut- and band-like nuclei (Fig. 1e). These morphological features suggested that PAC1 may be expressed by hematopoietic cells.

### Identification of PAC1^+^ cells

**Figure 2:**
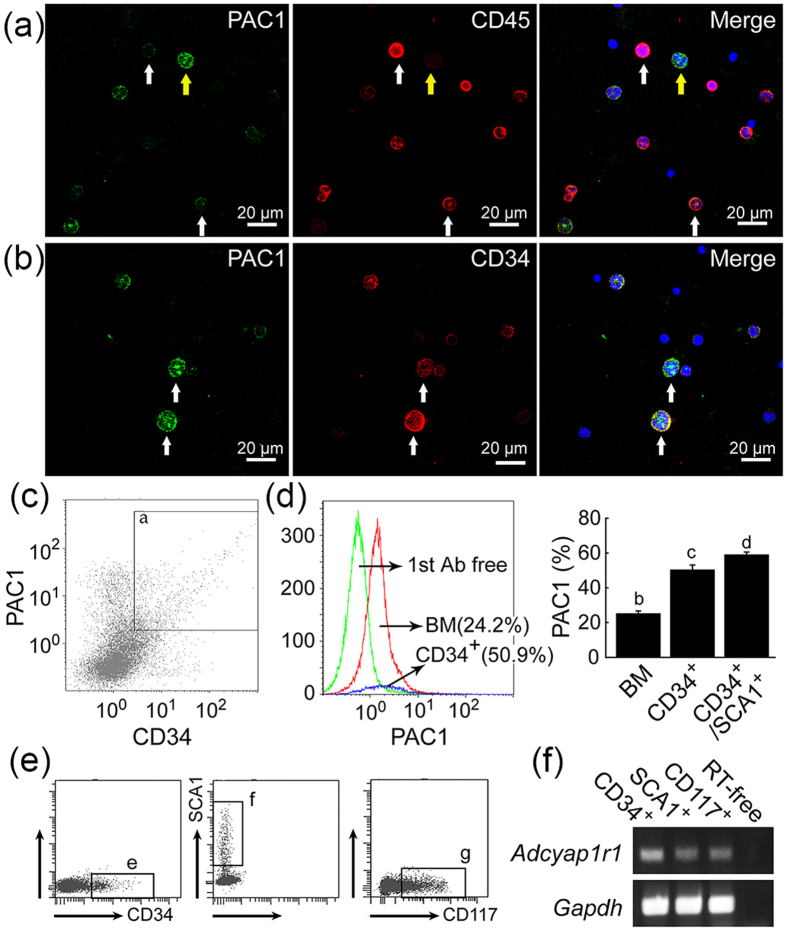
PAC1^+^ cells of stronger intensity are HPCs. (**a**) Most PAC1 positive cells (*green*) were also positive for CD45 (*red*, *arrows*). However, cells with stronger PAC1^+^ intensity were less likely to express CD45 (*yellow arrows*). (**b**) Most PAC1-positive cells in BM (*green*) were positive for CD34 (*red*). *Blue* in merged images represents nuclear staining with DAPI. Scale bar, 20 μm. (**c**) FCM analysis of PAC1 and CD34. (Gate *a*) cells expressing both PAC1 and CD34. (**d)** Percentage of PAC1^+^ cells was higher in CD34^+^ and CD34^+^/SCA1^+^ subpopulations than in total BM. All values are expressed as mean ± SEM (n = 3 per experimental group). Different lower-case letters in the Figures indicate statistically significant differences (*P* < *0.05*, Student-Newman-Keuls test). (**e**,**f**) CD34^+^ (gate *b*), SCA1^+^ (gate *c*) and CD117^+^ (gate *d*) subpopulations all expressed *adcyap1r1*. RT-free indicates the absence of reverse transcriptase (negative control). All results were normalized relative to the expression of the housekeeping gene *gapdh*.

The two types of PAC1^+^ cells could be differentiated by staining with antibodies against antigenic markers of hematopoietic cells (CD45) and hematopoietic progenitor cells (CD34) (Fig. 2). CD45 is a pan-hematopoietic cell marker, the expression of which increases as nucleated hematopoietic cells mature^33^,^34^. CD34 is a hematopoietic progenitor marker that is expressed by short-term HSCs and strongly expressed in multipotent progenitors (MPPs) and restricted progenitors, but is not expressed by long-term HSCs^35^,^36^,^37^. Although most (95.6 ± 3.0%) PAC1^+^ cells in smear sections were positive for CD45, greater intensity of PAC1^+^ staining was associated with weaker CD45 staining, and greater intensity of CD45^+^ staining was associated with little or no PAC1 expression. Staining of PAC1^+^ cells with antibodies to CD34 and CD117 (c-kit) showed a correlation betweenPAC1 and CD34 intensity (Fig. 2b), with cells strongly positive for PAC1 and CD34 also positive for CD117, another hematopoietic stem/progenitor marker (Supplementary Fig. S1C), suggesting that PAC1 might be expressed on immature hematopoietic cells. SCA1 is expressed by murine HSCs and MPPs^37^, but not by lineage-committed progenitors; thus CD34^+^/SCA1^+^ cells may represent populations enriched in short-term HSCs and MPPs. Flow cytometry (FCM) analysis showed that 24.2%, 50.9%, and 58.9% of nucleated BM, CD34^+^, and CD34^+^/SCA1^+^ cells, respectively, were positive for PAC1 (Fig. 2c,d). Moreover, cells sorted by positivity for CD34, SCA1 or CD117 were positive for *adcyap1r1* expression (Fig. 2e,f).

**Figure 3:**
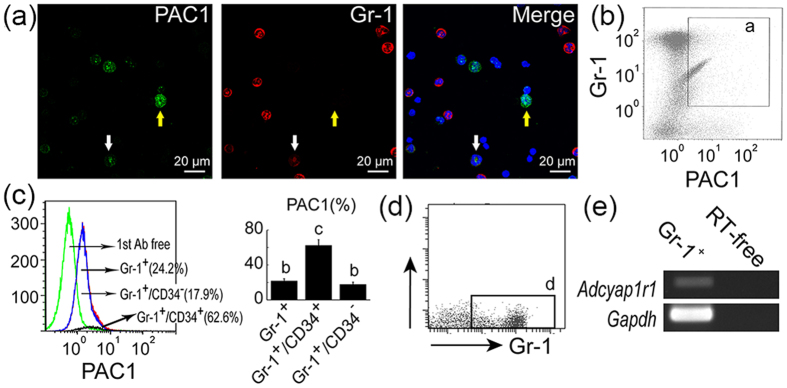
PAC1 expression on myeloid lineage cells. (**a**) PAC1^+^ cells (*green*) were positive for Gr-1 (*red*) in BM smear sections (*arrows*). Cells showing stronger expression of PAC1^+^ (*yellow arrows*) showed little or no expression of Gr-1, whereas cells showing weaker expression of PAC1^+^ cells showed definite expression of Gr-1 (*white arrows*). *Blue* in merged images represents nuclear staining with DAPI. Scale bar, 20 μm. (**b**) FCM analysis showing the numbers of cells (*a*) positive for both PAC1 and Gr-1. (**c**) Percentages of PAC1^+^/Gr-1^+^ cells in BM was higher in Gr-1^+^/CD34^+^ than in Gr-1^+^/CD34^−^ subpopulations. All values are expressed as mean ± SEM (n = 3 per experimental group). Different lower-case letters in the Figures indicate significant differences (*P* < 0.05, Student-Newman-Keuls test). (**d,e**) Gr-1^+^ (gate *b*) cells express *adcyap1r1*. RT-free represents absence of reverse transcriptase (negative control). All results were normalized relative to the expression of the housekeeping gene *gapdh*.

To identify weaker intensity PAC1^+^ cells, cells were co-stained with antibodies to PAC1 and the lymphoid (CD45R), erythroid (TER119), and myeloid (Gr-1) lineage cell markers. Cells weakly positive for PAC1 were also positive for Gr-1 (Fig. 3a), whereas no or few cells were positive for CD45R and TER119. RT-PCR analysis also showed that sorted Gr-1^+^ cells weakly expressed *adcyap1r1* (Fig. 3e). As promyelocytes have a Gr-1^+^/CD34^+^ phenotype and mature granulocytes have a Gr-1^+^/CD34^−^ phenotype^38^, FCM analysis confirmed that, of the 24.2% of BM cells that were Gr-1^+^/PAC1^+^, 62.6% were Gr-1^+^/CD34^+^ and 17.9% were Gr-1^+^/CD34^−^ (Fig. 3c,d).

### *Adcyap1* ^−/−^ mice had fewer HPCs and an altered cell cycle

**Figure 4:**
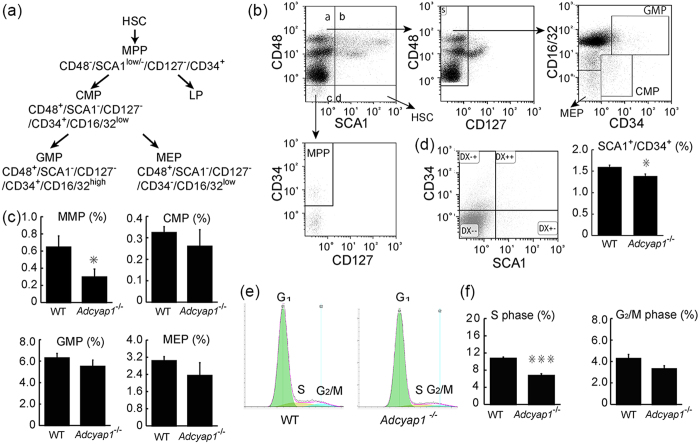
HPC subpopulations and cell cycle in a*dcyap1*^*−/−*^ mice. (**a**) Hierarchic and related markers of HPCs. (**b**) Representative FCM patterns used in the present experiments. HPC subpopulations included MPPs, CMPs, GMPs and MEPs. (**c**) Quantification of HPC subpopulations in BM of *adcyap1*^*−/−*^ and wild-type (WT) littermates. (**d**) CD34^+^/SCA1^+^ enriched HPC (gate DX++) frequencies in BM of *adcyap1*^*−/−*^ and WT mice. (**e**) Representative FCM analysis of intercellular PI staining of BM nucleated cells from WT and *adcyap1*^*−/−*^ mice. The percentages of cells in G_1_ (*green*), S (*yellow*) and G_2_/M (*blue*) phases were counted. (**f**) Quantified analysis showing that the percentage of cells in S phase was lower in *adcyap1*^*−/−*^ than in WT mice. All values are expressed as mean ± SEM (n = 3 per experimental group). *P < 0.05, **P < 0.01 (Student’s *t* test).

To determine the effect of PACAP on hematopoietic cells, HPCs of BM were compared in *adcyap1*^*−/−*^ and wild-type mice. In general, HSCs sit atop a hierarchy of progenitors and develop into MPPs (CD48^−^/SCA1^low/−^/CD34^+^/CD127^−^) (Fig. 4a), which, in turn, differentiate into lymphoid progenitors (LPs) and common myeloid progenitor (CMPs, CD48^+^/SCA1^−^/CD127^−^/CD34^+^/CD16/32^low^). CMPs further differentiate into granulocyte-macrophage progenitors (GMPs, CD48^+^/SCA1^−^/CD127^−^/CD34^+^/CD16/32^high^) and megakaryocytic/erythroid progenitors (MEPs, CD48^+^/SCA1^−^/CD127^−^/CD34^−^/CD16/32^low^)^35^,^39^,^40^. GMPs finally produce myeloid cells. The percentage of MPPs was significantly lower in the BM of *adcyap1*^*−/−*^ than of wild-type mice (0.3% vs. 0.6%, *P* < 0.05; Fig. 4b,c). In addition, the percentage of SCA1^+^/CD34^+^ enriched HPCs tended to be lower in *adcyap1*^*−/−*^ mice (Fig. 4d).

To determine the cell mechanisms associated with these differences, the cell cycle profile in BM of *adcyap1*^*−/−*^ and wild-type mice was assessed by propidium iodide (PI) nuclear staining. The percentage of cells in S-phase was significantly lower in the BM of *adcyap1*^*−/−*^ (6.7%) than of wild-type mice (10.7%, *P* < 0.01), whereas the percentage of cells in G_2_/M-phases was similar (3.7% vs. 4.7%) (Fig. 4e,f).

### PACAP38 augmented the proliferation of BM derived HPCs via PACAP receptor

**Figure 5:**
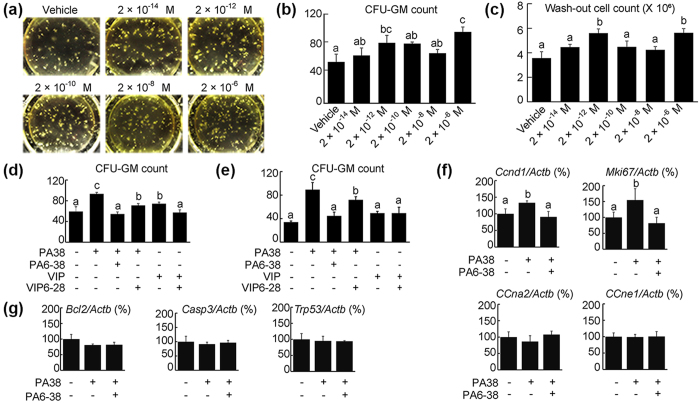
The PACAP-PAC1 pathway contributes to the proliferation of BM-derived CD34^+^/SCA1^+^ HPCs. (**a**) Representative images of colonies formed by HPC-derived cells in semi-solid culture after treatment with vehicle or PACAP38 (PA38, 2 × 10^−14^ to 2 × 10^−6^ M). (**b,c**) PACAP38 increased the numbers of CFU-GM colonies (**b**) and cells (**c**), as shown by a bell shaped curve with two putative peaks at 2 × 10^−12^ and 2 × 10^−6^ M. (**d**) The PACAP38 (PA38, 2 × 10^−6^ M) induced increase in CFU-GM colonies was blocked completely by PACAP6-38 (PA6-38) and partially by VIP6-28 (1 × 10^−5^ M each). VIP (2 × 10^−6^ M) increased CFU-GM and was blocked by VIP6-28. (**e**) A lower concentration of PACAP38 (2 × 10^−12^ M) increased CFU-GM, an effect blocked completely by PACAP6-38 (PA6-38) and partially by VIP6-28 (1 × 10^−11^ M each). However, VIP (2 × 10^−12^ M) did not increase the number of CFU-GM colonies. (**f,g**) Cell cycle (**f)** and apoptosis (**g**) related gene expression in SCA1^+^/CD34^+^ HPCs following treatment with PACAP38 (PA38). **(f**) PACAP38 (PA38, 2 × 10^−12^ M) increased the levels of expression of *ccnd1* and *mki67*, but not of *ccna2* and *ccne1*. These effects were blocked by treatment with PACAP6-38 (PA38, 1 × 10^−11^ M). (**g**) PACAP38 (PA38, 2 × 10^−12^ M) and PACAP6-38 (PA6-38, 1 × 10^−11^ M) did not alter the level of expression of selected apoptosis-related genes (*bcl2, casp3,* and *trp53*). All values are expressed as mean ± SD (n = 3 per experimental group). Different lower-case letters in the Figures indicate significant differences (*P* < 0.05, Student-Newman-Keuls test).

To assess the direct effect of PACAP on hematopoiesis, 2,000 CD34^+^/SCA1^+^ cells in semi-solid culture were incubated in the presence or absence of PACAP38 for 10 days, and the number of colonies was determined. Treatment with PACAP38 significantly increased the number of CFU-GM progenitors colonies, with peaks at 2 × 10^−12^ and 2 × 10^−6^ M, compared with vehicle-treated cells (55.6 ± 10.4) (Fig. 5a,b). Similar results were obtained by counting the total number of cells in collected colonies (Fig. 5c). The effect of PACAP38 on colony formation was antagonized by incubation with five-fold higher concentrations of PACAP6-38 and partially antagonized by five-fold higher concentrations of VIP6-28. PACAP6-38 is an antagonist for PAC1 and VPAC2, and VIP6-28 is an antagonist for VPAC1 and VPAC2^41^. Although 2 × 10^−6^ M VIP, which binds mainly to VAPC1 and 2, also increased the number of colonies and was antagonized by VIP6-28, a lower concentration (2 × 10^−12^ M) of VIP did not increase the number of colonies (Fig. 5d,e; Supplementary Fig. S2A). Similar results were obtained using CD34^+^ singly-positive cells, in that 2 × 10^−6^ M PACAP38 significantly increased the number of CFU-GM compared with vehicle, with this effect of PACAP38 antagonized by five-fold higher concentrations of PACAP6-38 (Supplementary Fig. S3A).

As cell number represents the balance between cell proliferation and cell death, we assessed whether exogenous PACAP38 modulated the expression of genes associated with the cell cycle and affected apoptosis. Treatment of CD34^+^/SCA1^+^ cells in semi-solid culture with PACAP38 (2 × 10^−6^ and 2 × 10^−12^ M) for 3 days significantly increased the expression of *ccnd1* and *mki67* mRNAs, effects antagonized by co-treatment with PACAP6-38 (1 × 10^−5^ and 10^−11^ M, respectively) (Fig. 5f, Supplementary Fig. S2B). PACAP38, however, did not affect the expression of *ccna2* and *ccne1*, and the apoptosis-related genes *bcl2*, *casp3* and *trp53* (Fig. 5G and Supplementary Fig. S2C). Similar results were confirmed in semi-solid cultures of cells singly positive for CD34^+^ (Supplementary Fig. S3B,C).

### PACAP-containing paravertebral sympathetic ganglia innervated into murine BM

**Figure 6:**
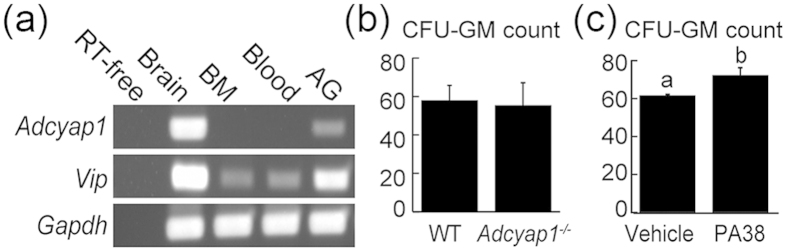
Expression of the PACAP-encoding gene (*adcyap1*) in mouse BM. (**a**) Expression of *adcyap1* and *vip*, encoding PACAP and VIP, respectively, in BM, blood and adrenal glands (AG) of mice. RT-free indicates the absence of reverse transcriptase (negative control), and brain sample was used as a positive control. Results were normalized relative to the expression of *gapdh. Vip* was expressed in all tissues, whereas little *adcyap1* expression was observed in BM and blood. (**b**) CFU-GM colonies after 10 days, following culture of nucleated BM cells of wild-type (WT) and *adcyap1*^*−/−*^ mice. No difference in colony formation was observed. (**c**) Exogenous PACAP38 (PA38, 2 × 10^−6^ M) enhanced CFU-GM generation from BM cells. All values are expressed as mean ± SD (n = 3 per experimental group). *P < 0.05 (Student’s *t* test).

Although these results indicated that endogenous and exogenous PACAP may contribute to hematopoiesis via PAC1, the source of PACAP in BM was unclear. We therefore assessed whether PACAP was produced in BM via an autocrine/paracrine system. The levels of expression of *adcyap1* and *vip* mRNAs were determined in the brain, BM, blood and adrenal glands (Fig. 6a). As a positive control, brain tissue showed abundant expression of both *adcyap1* and *vip*. In agreement with previous findings, BM, blood and adrenal glands were also positive for *vip*^42^. Although *adcyap1* was expressed in adrenal glands, little or no expression was observed in BM and blood. These results were confirmed by semi-solid culture. BM-nucleated cells from *adcyap1*^*−/−*^ and wild-type mice showed no difference in CFU-GM colony formation (Fig. 6b). In contrast, PACAP38 treatment significantly increased the number of BM cells compared with vehicle (Fig. 6c).

**Figure 7:**
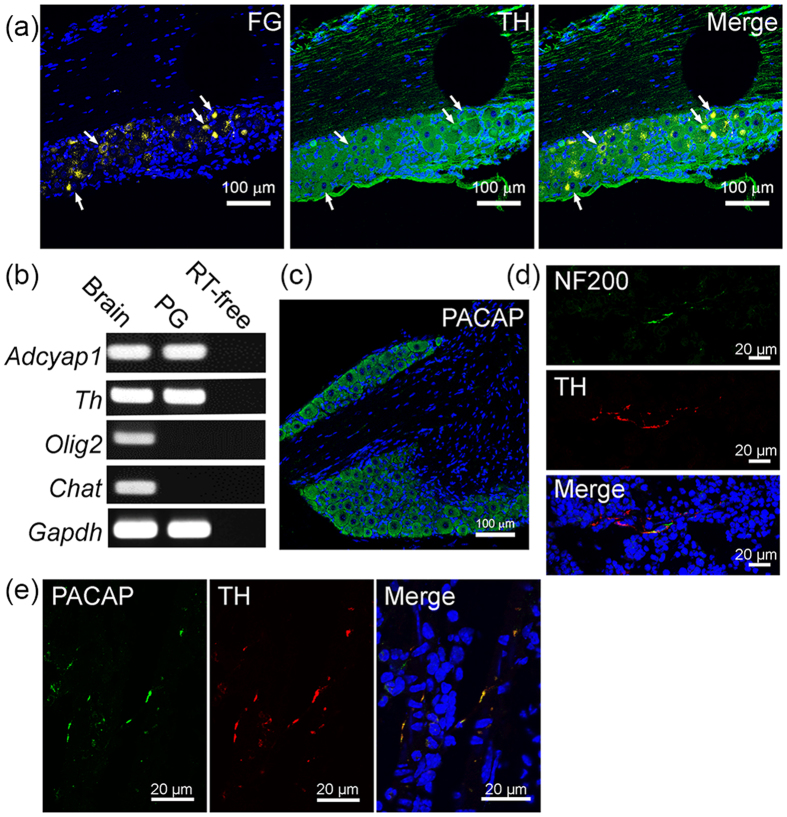
Paravertebral sympathetic innervation is a potential source of PACAP in BM. (**a**) Fluorogold was injected into murine left tibial BM for 7 days. Ipsilateral paravertebral ganglia positive for liposome-like gold signals were observed (*left*, *white arrow*). Most of the cells in these ganglia were positive for expression of TH (*middle*), with some of these cells also positive for Fluorogold (*right*). **(b**) RT-PCR expression of *adcyap1, th, olig2* and *chat* mRNAs in brain and paravertebral ganglia (PG) (**c**) Sympathetic trunk sections were stained with DAPI (*blue*) and anti-adcyap1 antibody (*green*). (**d)** Co-staining of BM frozen sections with anti-NF 200 (*green*) and anti-TH (*red*) antibodies and DAPI (*blue*). **(e)** Triple-labeled staining with anti-PAC1 adcyap1 (*green*) and anti-TH (*red*) antibodies and DAPI (*blue*). Scale bars, 100 μm in (**a,b**); 20 μm in **(d,e**).

We examined next whether PACAP in BM was derived from peripheral nerve innervations. To determine the ganglia that innervate into BM, we injected a retrograde neural tracer, Fluorogold, into the left tibia of mice (Supplementary Fig. S4A,B), finding Fluorogold-labeled neural bodies in the ganglia on the retroperitoneal body trunk (Th12 to S2 levels) after seven days. Fluorogold signals were observed in the BM cavity, with no obvious leakage into muscle and around the tissues immediately after dye injection (Supplementary Fig. S4C). Fluorogold-labeled signals were observed on the left paravertebral ganglia at the L3 to L5 levels (Supplementary Fig. S4D–F). These Fluorogold-labeled cells were positive for the sympathetic nerve marker, tyrosine hydroxylase (TH) (Fig. 7a). Assays of collected L3-L5 ganglia for expression of mRNAs encoding PACAP and neural makers showed that these ganglion samples expressed abundant amounts of *adcyap1* (Fig. 7b) and *th* mRNAs, but not *olig2*, a marker of motor neurons, or *chat*, a marker of parasympathetic neurons. Immunostaining for pre-pro PACAP also confirmed the localization of PACAP in the paravertebral ganglia (Fig. 7c). Finally, prepro-PACAP-positive sympathetic nerve fibers were present in the tibal BM, as approximately 80% of tibial BM cells positive for the neurofilament 200 (NF200) were also positive for TH (Fig. 7d), and prepro-PACAP co-localized with TH (Fig. 7e).

## Discussion

PACAP is a multi-functional peptide originally isolated from the hypothalamus and found to contribute to the development of cells of the ectodermal lineage^18^,^19^,^20^,^43^. However, there was little evidence that PACAP contributed to the development of other lineages. This study showed a unique pattern of expression of PAC1 in nucleated mouse BM cells. Cells strongly positive for PAC1 were of relatively larger size and had oval nuclei, suggesting that these cells were hematopoietic stem/progenitor cells (HPSCs). In contrast, cells weakly positive for PAC1 were smaller in size with donut- and band-like nuclei, suggesting cells of the myeloid lineage. Further analysis supported these morphological observations, as the expression pattern of PAC1 resembled that of CD34, and cells strongly positive for CD45 and Gr-1 were weakly positive for PAC1. CD34 is a transmembrane phosphoglycoprotein regarded as a marker of tissue-specific stem cells, including muscle satellite cells (muscle stem cell), epidermal precursors and vascular endothelial progenitors. Although mouse long-term HSCs are negative for CD34^44^, CD34 is expressed by short-term HSCs and MPPs, becoming progressively downregulated on more mature cells^35^,^45^,^46^. *Cd34*-deficient mice were reported to have fewer HPCs in embryonic and adult tissues, and adult-derived progenitors appear to have a proliferation defect^47^. Taken together, these results suggest that PAC1 may be express by immature hematopoietic cell populations, especially HPCs. Our findings also suggest that PACAP may contribute to the development of the mesodermal lineage, as well as to odontoblast development, because infant *adcyap1* deficient (*adcyap1*^*−/−*^) mice have a of a thinner dentin layer than wild-type mice^22^. PACAP/PAC1 signaling has been reported to promote chondrogenesis by regulating calcineurin downstream^43^ and to inhibit megakaryocyte maturation through VPAC1 signaling via an anti-apoptotic pathway^24^,^25^.

In assessing role of PACAP in the development of HPCs *in vivo* and *in vitro*, we found fewer BM progenitor cell populations (MPPs, CMPs, GMPs, MEPs and LPs) in *adcyap1*^*−/−*^ mice than in wild-type mice. PACAP treatment of semi-solid cultures of SCA1^+^/CD34^+^ cells, enriched in short-term HSCs and MMPs, significantly increased the number of CFU-GM with two peaks. PACAP has been reported to bind to three PACAP/VIP receptors, PAC1, VPAC1 and VPAC2, with PAC1 having the highest affinity^48^. Treatment of cells with PACAP38 and the PAC1/VPAC2 antagonist PACAP6-38 or the VPAC1/VPAC2 antagonist and VIP6-28 showed recognized that the action of PACAP was mediated mainly via PAC1 and partially via VPAC2. At higher concentration, VIP, a homologue of PACAP, slightly increased the number of CFU-GM colonies number; as this reaction was antagonized by VIP6-28, it was likely mediated by VPAC2. For physiological reasons, however, these reactions may not occur in the BM.

To clarify the underlying mechanism by which PACAP increased the number of GFU-GM colonies, we assessed the effects of PACAP on apoptosis- and cell cycle-related genes. PACAP has been reported to contribute to the proliferation of NPCs and other cells in culture^49^,^50^ and to suppress apoptotic neural cell death in ischemic brains *in vivo* and in cerebellar granule neurons *in vitro*^2^,^3^,^4^,^51^. We found that PACAP treatment of BM cells increased the expression of the cell cycle-related genes, cyclin D1 and Ki67. The expression of cyclin D1 has been found to begin to increase during early G_1_-phase, peaks during S-phase, and decreases during M-phase^52^. Our results showed that the percentage of BM cells in S-phase was lower in *adcyap1*^*−/−*^ than wild-type mice. In contrast, PACAP had no effect on the expression of apoptosis-related genes. These results suggested that PACAP promoted HPC proliferation by accelerating the cell cycle, not by suppressing apoptosis.

There are three potential candidates that can provide PACAP to the BM: 1) an autocrine and/or paracrine system of hematopoietic cells and/or BM stromal compartments in the BM cavity, 2) circulating blood through the endocrine system, and 3) innervating neuronal terminals. We found that *adcyap1* was not expressed in BM and blood, and that the number of CFU-GM colonies in *adcyap1*^*−/−*^ and wild-type mice did not differ, suggesting that PACAP was not produced in the BM. To determine whether PACAP in BM was supplied by the nervous system, we used a retrograde neural tracer to identify neural bodies of ganglia that had innervated into the tibial BM, and determined paravertebral ganglia at levels L3 to L5. These ganglia were identified as sympathetic nerves and were observed to express prepro-PACAP mRNA and protein. We also observed prepro-PACAP expressing sympathetic nerve fibers in the BM cavity. PACAP modulation of other hematopoietic organs, including lymph node and thymus, has been reported mediated by sympathetic nerve innervations^13^. Although we could not exclude the possibility that PACAP in BM was supplied by the endocrine system through the circulation, our results suggest that PACAP may be a neurotransmitter supplied by sympathetic nerve innervations and that PACAP may play an important role in hematopoiesis.

We did not observe critical differences between *adcyap1*^*−/−*^ and wild-type mice in hematopoietic populations of BM. As impaired hematopoietic functions have not been observed in *adcyap1*^*−/−*^ mice under physiological conditions, it remains unclear whether PACAP contributes to the hematopoietic niche under homeostatic situations. However, *adcyap1* expression has been found to increase markedly during inflammation and disease^13^,^41^ and to alleviate inflammation, oxidative stress and apoptosis^13^,^26^,^53^. PACAP has been shown to reduce clinical symptoms and inflammation in mouse models of immune-based diseases, including rheumatoid arthritis, Crohn’s disease and multiple sclerosis^12^,^13^,^53^,^54^. The PACAP-VPAC2 system was found to promote BM-derived tolerogenic dendritic cells, mediated by a positive balance between Th2 and Th1 cytokines^42^,^48^,^49^,^55^. Indeed, both *adcyap1* and *adcyap1r1* were expressed in hBMSCs after stimulation with the pro-inflammatory cytokine IFNγ^26^. Although our results initially indicated that PACAP treatment commits cells to hematopoiesis, further studies are needed to clarify the activity of PACAP on hematopoietic cells under immune and inflammatory conditions.

## Conclusions

This study showed that PACAP contributes to hematopoiesis and cells of the mesodermal lineage. PAC1 expression may be hierarchically specific, was greater in CD34^+^ HPCs, and decreased and disappeared as cells of the myeloid lineage matured. This study also showed that the PACAP-PAC1 system promoted the proliferation of HPCs by enhancing cyclin D1 expression and increasing the number of cells in S-phase. Finally, this study found that PACAP in BM was supplied by innervating sympathetic neurons. These results suggested that PACAP may be a new candidate regulator of the hematopoietic niche.

## Materials and Methods

### Animals

C57/BL6 strain *adcyap1*^*−/−*^mice^16^ were maintained under specific pathogen-free conditions in the animal facility of Showa University. All experimental protocols were approved by the Institutional Animal Care and Use Committee of Showa University. All experiments were performed according to the guidelines of the Institutional Animal Care and Use Committee of Showa University.

### Bone marrow collection and cell sorting

Mice (n = 2–4) were anesthetized with sodium pentobarbital (50 mg/kg, i.p.) and their femurs and tibias obtained. The epiphyses of the bones were cut and immersed in 3.0 mL phosphate-buffered saline (PBS) in 50 mL conical tubes. Total BM was collected by centrifugation at 800 × g for 10 min. For cell sorting, BM cells were passed through an 18-gauge blunt needle to prepare single-cell suspensions. Red blood cells were lysed and the nucleated BM cells were incubated in 1.0 μg anti-mouse CD16/CD32 (BD Pharmingen) in 2.0% fetal bovine serum (FBS) for 10 min on ice to block non-specific and mouse Fc receptor binding. The cells were incubated with appropriate fluorochrome-labeled antibodies (Supplementary Table 1) for 45 min on ice, diluted to 3 × 10^6^ cells/mL and applied to a FACSAria (BD Biosciences) flow cytometer at 1,200 cells/sec. CD34, SCA1, CD117 and Gr-1 single positive cell populations were separated, and CD34^+^/SCA1^+^ and CD34^+^ cells were sorted for colony-forming unit (CFU) assays.

### RNA isolation and reverse transcription-polymerase chain reaction (RT-PCR)

Wild-type mice (n = 3) were anesthetized and whole blood was collected from the right ventricle of the heart. The brain, adrenal glands, spinal sympathetic trunk and hind limb bones were collected. Whole BM cells were collected and cell subpopulations sorted as above. Total RNA isolation and RT-PCR were performed as described^56^. The primers used in the experiments are listed in Supplementary Table S2. Reverse transcriptase-free (RT-free) reactions with RNA samples were used as negative controls in PCR reactions to show that genomic DNA was not amplified.

### Preparation of bone frozen sections and BM smears

Mice (n = 4) were anesthetized with sodium pentobarbital and perfused-fixed with PBS, followed by ice-cold 4% paraformaldehyde (PFA). The hind limb bones were decalcified for 3 days in 0.5 M EDTA (pH 7.4) at 4 °C and frozen blocks prepared. The bones were sectioned (5.0-μm thickness) on adherent film (Sigma-Aldrich, St. Louis, MO) according to Kawamoto method^57^. The films were placed onto glass slides with 5% gelatin. All sections were air-dried and kept at −30 °C until use.

To prepare BM smear sections, total BM was obtained from four mice and single cell suspensions were prepared as described above. The BM cells were resuspended in 1 mL PBS containing 5% bovine serum albumin and spread onto MAS-coated glass slides (Matsunami, Osaka, Japan). The slides were fixed with methanol, air dried, and stored at −30 °C until use.

### Immunofluorescent identification of PAC1 positive cells in BM

Sections were washed with PBS and 0.05% Tween-20 in PBS (PBST) and immersed in 0.25% Triton X-100 in PBS for 30 min to increase cell membrane permeability. After incubation in 10% normal horse serum (NHS, Vector Laboratories, Burlingame, CA)/PBST (blocking buffer), the slides were incubated with rabbit anti-PAC1 antiserum^58^ overnight at 4 °C and further incubated with Alexa Fluor 488-labeled anti rabbit IgG (1:400; Invitrogen, Carlsbad, CA) as a secondary antibody for 60 min. Nuclei were stained with 4′, 6-diamidino-2-phenylindole (DAPI). Decalcified BM sections were incubated with 0.1% Sudan Black B (Wako, Tokyo, Japan) in 70% ethanol for 30 min to quench auto-fluorescence^59^. To evaluate the specificity of PAC1 immunoreactions, antigen absorbance tests were performed. Anti-PAC1 antiserum was pre-incubated with a 10-fold concentration of antigen at 4 °C overnight, followed by centrifugation at 10,000 rpm for 10 min (antigen mixture). Tissue sections were subsequently incubated with antigen mixture or blocking buffer.

For multiple staining, the sections were blocked and incubated overnight at 4 °C with anti-PAC1 antiserum, along with non-labeled or fluorochrome-labeled antibodies against hematopoietic cell markers (Supplementary Table S1). After washing, the sections were incubated with secondary antibodies (Supplementary Table S1) and DAPI. The stained sections were observed and photographed with Nikon A1 laser confocal microscope (Nikon, Tokyo, Japan).

### Identification of PAC1-positive cells in BM by flow cytometry (FCM) analysis

Mononucleated BM cells (2 × 10^6^ cells) were incubated with rabbit anti-PAC1 antiserum for 30 min at RT and with Alexa FITC-labeled F(ab’)2 fragment of goat anti-rabbit IgG (Jackson Immunoresearch, West Grove, PA) for 30 min. These cells were incubated with Alexa 700-conjugated rat anti-mouse CD34 (BD Pharmingen), APC-conjugated rat anti-mouse Sca-1 and FITC-conjugated rat anti-mouse Gr-1 (eBioscinece, San Diego, CA), and applied to a Gallios (Beckman coulter) to detect fluorescence. All data were analyzed by Kaluza software (Beckman Coulter, La Brea, CA).

### CFU assay

BM mononuclear cells (280,000 cells), CD34^+^ cells (32,000 cells) or CD34^+^/SCA1^+^ cells (8,000 cells) were mixed with 4 mL semi-solid methylcellulose cultures supplemented with the recombinant cytokines mouse interleukin-3, mouse stem cell-derived factor 1, human interleukin-6, and human erythropoietin (M3434, StemCell Technologies, Vancouver, BC)^24^. The cell suspensions including PACAP38 (2 × 10^−14^ to 10^−6^ M, Peptide Institute, Osaka, Japan) were divided into 3 wells of a 6-well plate and cultured for 10 days at 37 °C and 95% humidity in an incubator containing 5% CO_2_ in air. In the antagonist experiments, a five-fold higher concentration of PACAP 6–38 (Peptide Institute) or VIP 6–28 (Abcam, Cambridge, CA) was added to the medium 60 min before addition of PACAP or VIP. The number of CFU-GM colonies was counted in a 2 × 2 cm area of STEMgrid™-6 (StemCell Technologies) under light microscopy (Nikon Eclipse TE300, Tokyo, Japan) and photographed with a Stylus TG-3 camera (Olympus, Tokyo, Japan) in microscopy mod. Cells in the methylcellulose-based media were repeatedly washed and centrifuged, and finally resuspended in 1.0 mL PBS for cell counting using an automated cell counter (TC20^TM^, Bio-Rad, Hercules, CA).

### Real time RT-PCR

The expression of genes associated with the cell cycle and apoptosis was assessed by real-time PCR using SYBR Premix Ex Taq II reagent (TaKaRa) and an ABI PRISM 7900 system (Applied Biosystems), as described^60^. The primers, listed in Supplementary Table S2, were purchased from Eurofins Genomics (Huntsville, AL).

### Determination of cell cycle

Cell cycle was determined by nuclear staining with PI. Briefly, suspensions of single cells were fixed in 70% ethanol at −20 °C for 4 hrs and incubated with 10 μg ribonuclease A at 37 °C for 30 min to hydrolyze RNA. The cells were incubated with 2 μg/mL PI (BD Pharmingen) at 4 °C for 30 min, and analyzed immediately by Gallios flow cytometry and Flowjo software (Tree Star, Ashlan, OR).

### Retrograde neuronal tracer injection into bone marrow

Mice were anesthetized by inhalation of 4.0% sevoflurane, with anesthesia maintained with 3.0% sevoflurane. Under aseptic conditions, the mice were placed on their backs with the left rear knee bent (Supplementary Fig. 4A,B). A small hole was drilled in the left tibia onto the proximal epiphysis. The BM cavity of each tibia was injected with 0.25 μL of 4% Fluorogold (Biotium Inc., Hayward, CA) at a rate of 0.125 μL/min using an ultra-micro pump (World Precision Instruments, Sarasota, FL). The burred hole was sealed with bone wax (Ethicon, Cincinnati, OH), and the muscles and skin surrounding the bone were sutured. Three animals were each sacrificed by perfusion-fixation on the day of dye-injection and for seven days thereafter. The body trunk of each mouse was collected from the Th12 to the S2 level and excess muscular tissues around the spinal canal trimmed. Each trunk was decalcified by immersion for five days in 20% sucrose/10% EDTA in 0.2 M PB (pH 7.2) followed by embedding with 20% sucrose:OCT compound (=1:1) in liquid nitrogen-chilled 2-methyl butane.

### Characterization of BM innervating neurons

To assess Fluorogold-positive cells, 5.0-μm thick frontal sections of the body trunk, prepared as described^57^, were examined by fluorescence microscopy using an ultraviolet excitation filter (Olympus AX-70, Olympus, Tokyo, Japan). After determining the regions of the sympathetic trunks that were labeled with Fluorogold, the spinal cord and sympathetic trunks were carefully removed from the spinal canal without decalcification, and frozen sections were prepared. These sections were incubated with sheep anti-TH antibody (Chemicon, Temecula, CA) at 4 °C overnight, followed by incubation with Alexa 488-labeled goat anti-sheep antibody for 90 min. Sections not labeled with Fluorogold were incubated with rabbit anti-adcyap1 (pre-pro-PACAP) polyclonal antibody (Antibody Verify, Las Vegas NV), which binds to prepro-PACAP. BM frozen sections were incubated with sheep anti-TH antibody plus rabbit anti-NF200 (Sigma-Aldrich) or rabbit anti-adcyap1 polyclonal antibody.

### Statistical analysis

*In vivo* experimental data are expressed as mean ± SEM and *in vitro* experimental data as mean ± SD. Statistical comparisons were made by one-way ANOVA followed by Student-Newman-Keuls tests for multiple group comparisons. Results in wild-type and *adcyap1*^*−/−*^ mice were compared using two-tailed Student’s *t* tests. *P* < 0.05 was considered statistically significant.

## Additional Information

**How to cite this article**: Xu, Z. *et al.* Pituitary adenylate cyclase-activating polypeptide (PACAP) contributes to the proliferation of hematopoietic progenitor cells in murine bone marrow via PACAP-specific receptor. *Sci. Rep.* **6**, 22373; doi: 10.1038/srep22373 (2016).

## Supplementary Material

Supplementary Information
