# Supplementary material for: Pituitary adenylate cyclase-activating polypeptide (PACAP) contributes to the proliferation of hematopoietic progenitor cells in murine bone marrow via PACAP-specific receptor
Source: Sci Rep. 2016 Feb 29;6:22373. doi: 10.1038/srep22373 (PMC4772629; doi:10.1038/srep22373)
Supplement: Supplementary Information [file srep22373-s1.doc]

**Pituitary adenylate cyclase-activating polypeptide (PACAP) contributes to the proliferation of hematopoietic progenitor cells in murine bone marrow via PACAP-specific receptor**

Zhifang Xu^1,2,7^, Hirokazu Ohtaki^1✳^, Jun Watanabe^1,3^, Kazuyuki Miyamoto^1^, Norimitsu Murai^1^, Shun Sasaki^1^, Minako Matsumoto^1^, Hitoshi Hashimoto^4^, Yutaka Hiraizumi^5^, Satoshi Numazawa^2^, Seiji Shioda^1,6,✳^

**Supplementary Figures:**

**
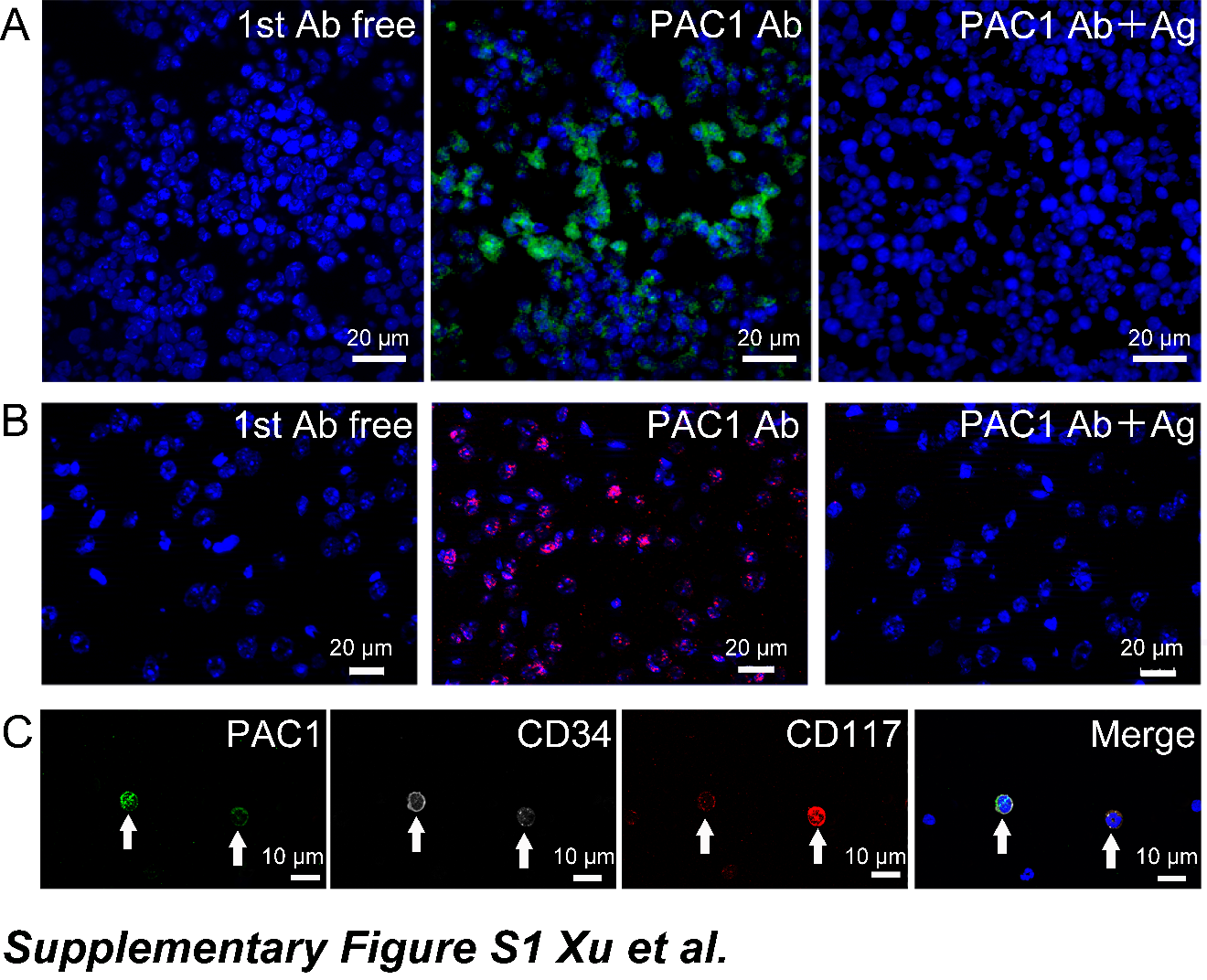
**

**Supplementary Figure S1.** **Distribution of PAC1 in BM.** (A-B) Absorption test of anti-PAC1 antibody. Immunohistochemical staining for PAC1 of frozen sections of BM (A) and brain (B). Sections were incubated without (1st Ab free) and with (PAC1 Ab) primary antibody, or with antibody plus antigen (PAC1 Ab + Ag), followed by incubation with secondary antibody. (C) PAC1 (*green*) expression on cells doubly positive for CD34 (*white*) and CD117 (*red*). The *blue* color represents nuclear staining with DAPI (*blue*). Co-expressions are showed as white arrows, Scale bar, 20 μm in A and B, 10 μm in C.

**
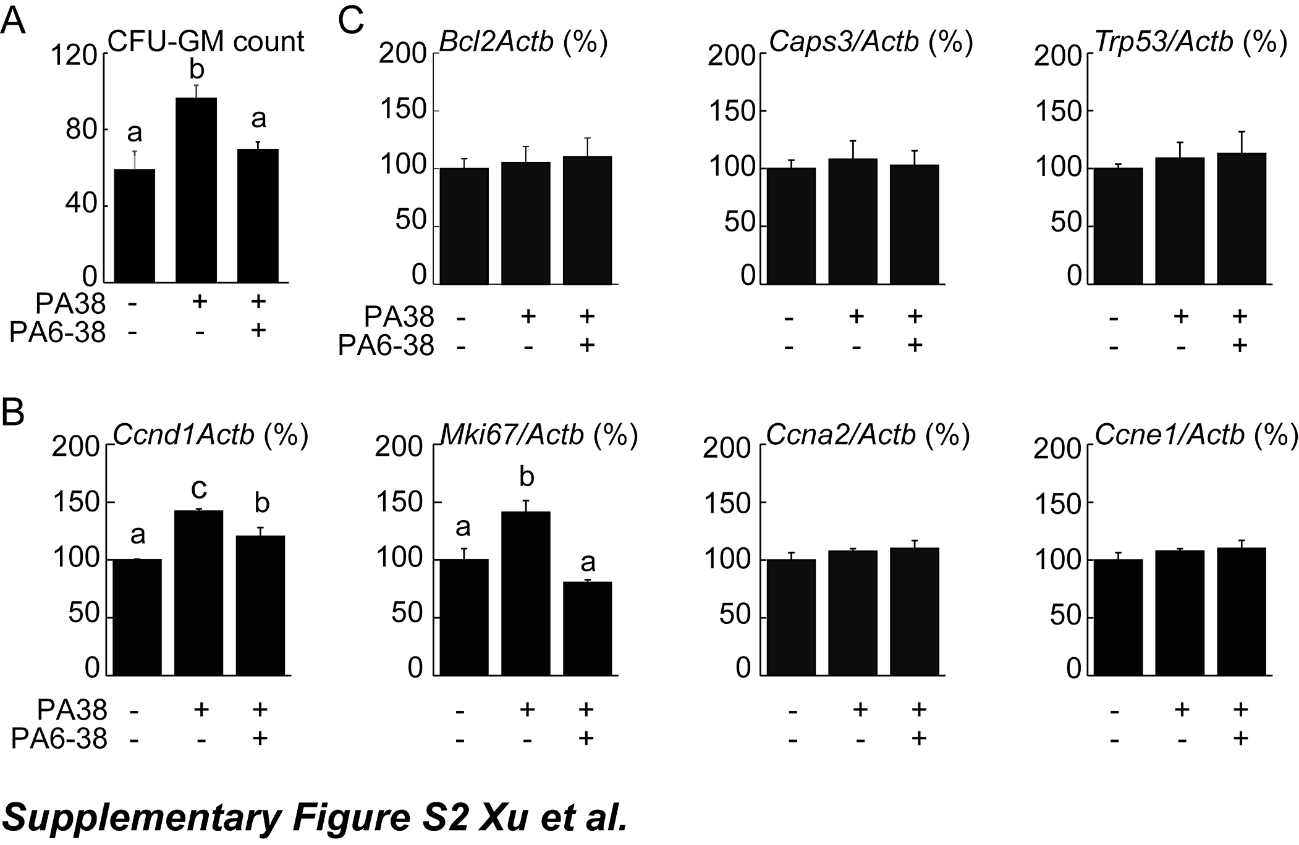
**

**Supplementary Figure S2.** **PACAP-PAC1 pathway stimulates the proliferation of BM-derived CD34^+^/SCA1^+^ HPCs.** (A) The PACAP38 (PA38, 2 × 10^-10^ M)-induced increase of CFU-GM number was abolished by co-treatment with PACAP6-38 (PA6-38, 1 × 10^-9^ M). Mean ± SD (n = 3, *P* < 0.05, Student-Newman-Keuls test); common letters indicate a lack of statistical significance. (B-C) qRT-PCR analysis of the expression levels of genes associated with the cell cycle (B, *ccnd1, mki67, ccna2, ccne1*) and apoptosis (C, *bcl2, casp3, trp53*) following treatment of SCA1^+^/CD34^+^ cells with PACAP38 (PA38, 2 × 10^-6^ M) in the presence or absence of PACAP6-38 (PA6-38, 1 × 10^-5^ M) for 3 days. Values are reported as mean ± SD (n = 3, Student-Newman-Keuls test); common letters indicate a lack of statistical significance.

**
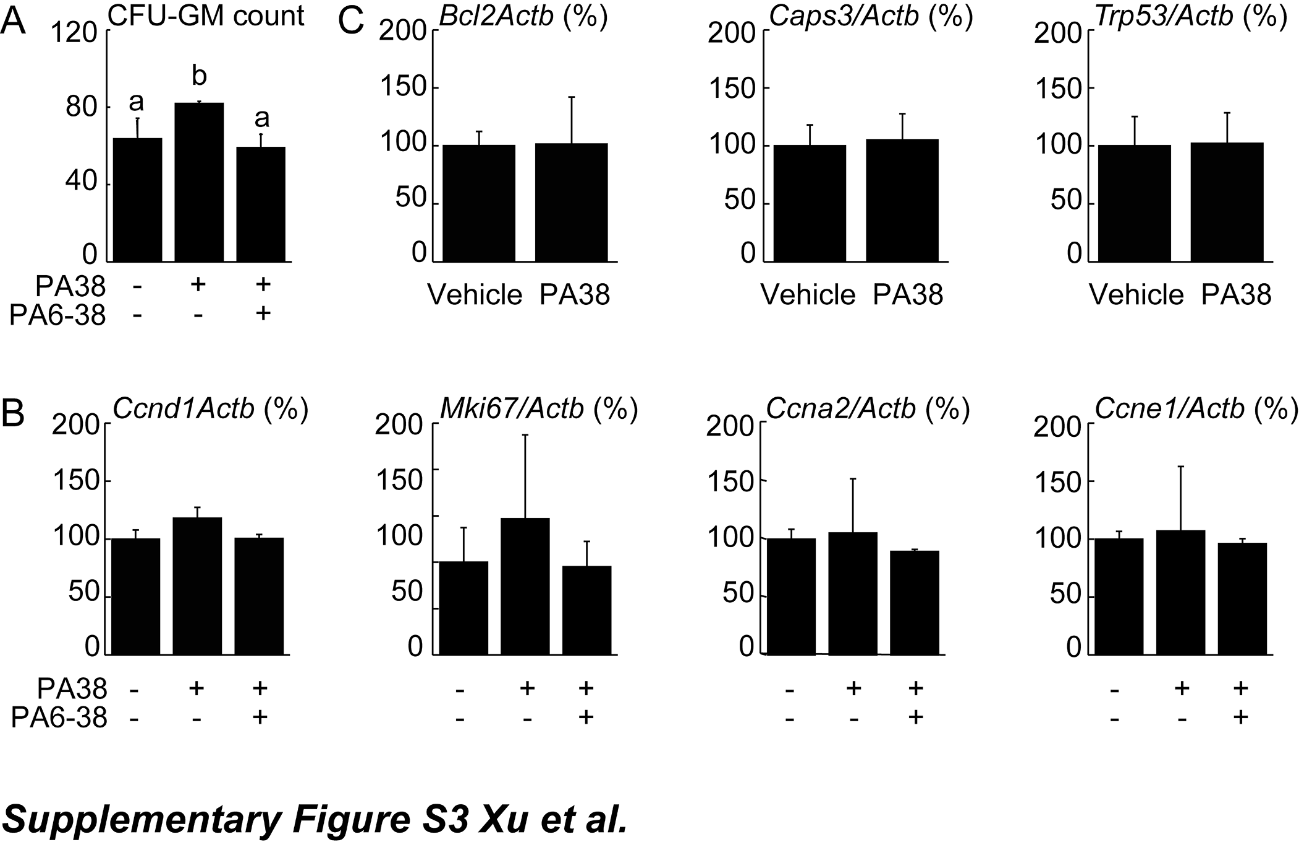
Supplementary Figure S3.** **PACAP-PAC1 pathway stimulates the proliferation of BM-derived CD34^+^ HPCs.** (A) PACAP38 (PA38, 2 × 10^-6^ M) induced increase of CFU-GM number was blocked by co-treatment with PACAP6-38 (PA6-38, 1 × 10^-5^ M). Mean ± SD (n = 3, Student-Newman-Keuls test); common letters indicate a lack of statistical significance. (B-C) qRT-PCR analysis of the expression levels of genes associated with the cell cycle (B, *ccnd1, mki67, ccna2, ccne1*) and apoptosis (C, *bcl2, casp3, trp53*) following treatment of CD34^+^ cells with PACAP38 (PA38, 2 × 10^-6^ M) in the presence or absence of PACAP6-38 (PA6-38, 1 × 10^-5^ M) for 3 days. Values are reported as mean ± SD (n = 3, Student-Newman-Keuls test), common letters indicate a lack of statistical significance.

**
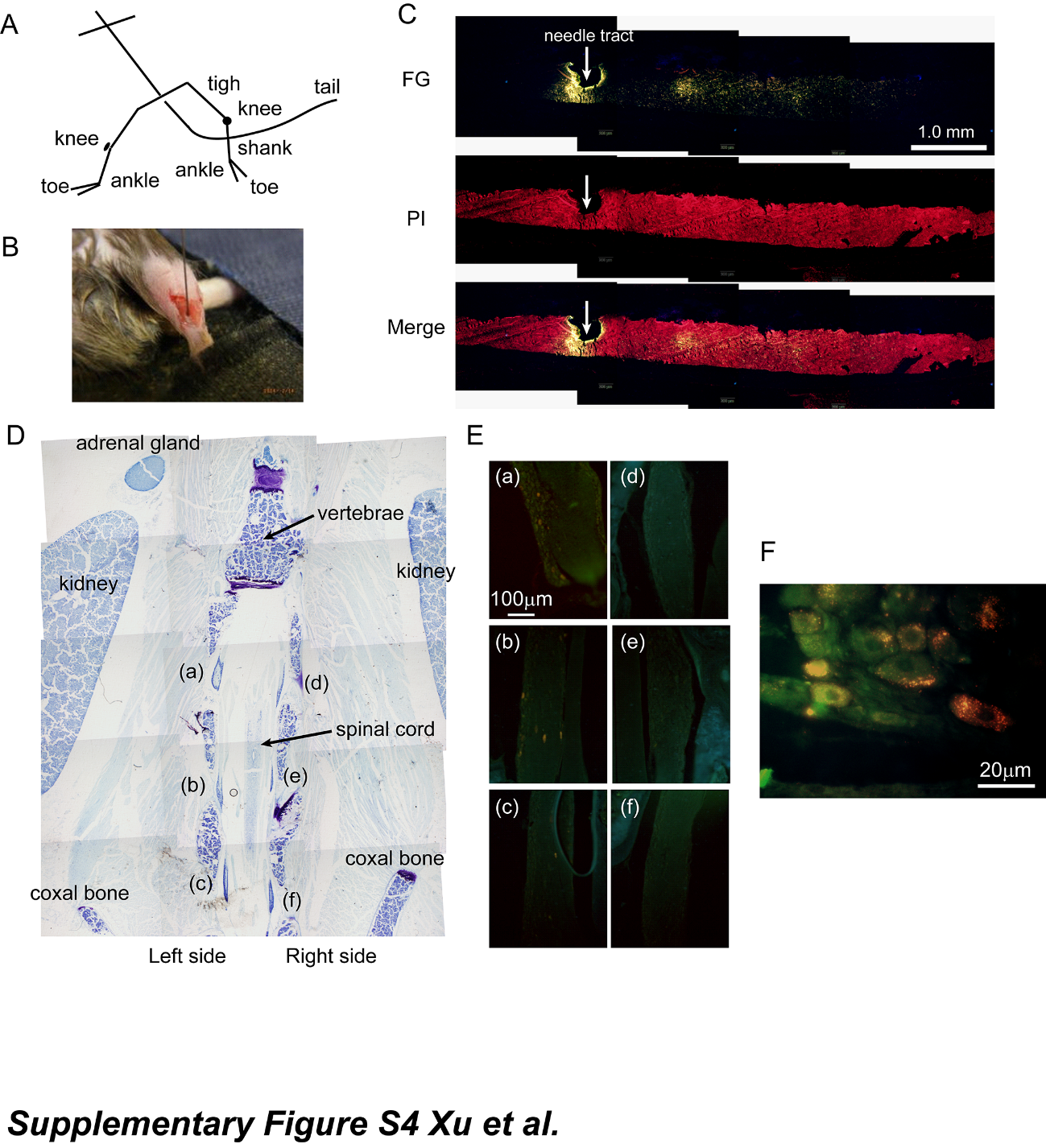
**

**Supplementary Figure 4.** **Tibial Fluorogold injection traced innervating ganglia into the tibia.** Illustrated bodily posture (A) and an image (B) of mice of Fluorogold injection into left tibia. (C) Visualization of Fluorogold signal (FG) immediately after dye injection. FG signals were observed around needle tract and some signals were extended in BM cavity. Many nucleated BM cells were labeled by nucleic staining PI (*red*), and merged image showing most of FG dye were successfully injected into tibial BM cavity. (D) A typical toluidine blue staining of retroperitoneal body trunk from Th12 to L6 levels. (a) to (f) are paravertebral ganglia at the lumber level 3 (a and d), 4 (b and e) and 5 (c and f). (E) Seven days after dye injection, FG-positive signals were only observed on the left side of L3-5 paravertebral ganglia by UV-laser of AX70 microscopy (Olympus) (a - c). (F) Higher magnification of FG-positive cells in L3 paravertebral ganglia. Scale bars, 1.0 mm in C; 100 μm in E; 20 μm in F.

**Supplementary Table S1. List of first and second** antibodies

| **First Antibodies** | **Dye label** | **Clone #** | **Host** | **Company** | **Catalog #** | **Application** | **Folds** |
| --- | --- | --- | --- | --- | --- | --- | --- |
| Anti-CD34 | Alexa Fluor 700 | RAM34 | rat | BD pharmingen | 560518 | FCM^1^/IF^2^ | 50/200 |
| Anti-CD45 | PE | 30-F11 | rat | eBioscience | 12-0451 | FCM/IF | 500/200 |
| Anti-CD117 | PE | 2B8 | rat | eBioscience | 12-1171 | FCM/IF | 200/200 |
| Anti-Gr-1 (Ly-6G) | FITC | RB6-8C5 | rat | eBioscience | 11-5931 | FCM | 200 |
| Anti-SCA1 | APC | D7 | rat | eBioscience | 17-5981 | FCM | 300 |
| Anti-Adcyap1 | Purified | Polyclonal | rabbit | Antibody Verify | AAS16419c | IF | 400 |
| Anti-CD16/32 | Purified | 2.4G2 | rat | BD pharmingen | 553141 | FCM | 50 |
| Anti-Gr-1(Ly-6C and Ly-6G) | Purified | RB6-8C5 | rat | BD pharmingen | 557445 | IF | 200 |
| Anti-NF-200 | Purified | Polyclonal | rabbit | Sigma | N-4142 | IF | 1000 |
| Anti-PAC1 | Purified | Polyclonal | rabbit | *Suzuki el al, 2003* |  | FCM/IF | 100/400 |
| Anti-TH | Purified | Polyclonal | sheep | Chemicon | AB1542 | IF | 500 |
|  |  |  |  |  |  |  |  |
| **Second Antibodies** | **Dye label** | **Clone #** | **Host** | **Company** | **Catalog #** | **Application** | **Folds** |
| Anti-rabbit IgG (H+L) | Alexa Fluor 488 |  | goat | Invitrogen | A11034 | IF | 400 |
| Anti-rabbit IgG (H+L) | Alexa Fluor 546 |  | goat | Invitrogen | A11035 | IF | 400 |
| Anti-sheep IgG (H+L) | Alexa Fluor 488 |  | donkey | Invitrogen | A11015 | IF | 400 |
| Anti-sheep IgG (H+L) | Alexa Fluor 546 |  | donkey | Invitrogen | A21098 | IF | 400 |
| Anti-rabbit IgG ( F(ab')2 Fragment) | FITC |  | goat | Jackson immunoresearch | 111-096-144 | FCM | 100 |

^1^FCM: flow cytometry, ^2^IF: immunofluorescent staining, ^3^PE: phycoerythrin, ^4^FITC: fluorescein isothiocyanate, ^5^APC: allophycocyanin

**Supplementary Table S2. List of primers**

| **Genes** | **Accession Numbers** | **Forward Primers** | **Reverse Primers** | **Product size (bp)** |
| --- | --- | --- | --- | --- |
| *Actb*^1^ | NM_007393 | catccgtaaagacctctatgccaac | atggagccaccgatccaca | 171 |
| *Adcyap1*^2^ | NM_009625 | ccgaaaacaaatggctgtcaag | ctgtgcattctctagtgcttca | 275 |
| *Adcyap1r1*^3^ | NM_001025372.2 | ggaatgcaaagctgtcatggtt | caagatggagatgaggatgggg | 326 |
| *Bcl2*^4^ | NM_009741 | gatgactgagtacctgaaccgg | gcatatttgtttggggcaggtt | 223 |
| *Casp3*^5^ | NM_001284409.1 | atacatgggagcaagtcagtgg | aatgtctctctgaggttggctg | 173 |
| *Ccna2*^6^ | NM_009828 | cccagtacttcctgcacctg | gttgtgccaatgactcaggc | 189 |
| *Ccnd1*^7^ | NM_007631 | tgcgtgcagaaggagattgt | cttcttcaagggctccaggg | 150 |
| *Ccne1*^8^ | NM_007633 | tatggtgtcctcgctgcttc | gggtctggatgttgtgggag | 202 |
| *Chat*^9^ | NM_009891.2 | tttgaaggagcaggtgaccc | acggcatcagagtgtcgttt | 280 |
| *Gapdh*^10^ | NM_008084 | gctacactgaggaccaggttgt | ctcctgttattatgggggtctg | 306 |
| *MKi67*^11^ | NM_001081117 | catgcaaactctccctgtacca | tcgctgaattggaaagtacgga | 217 |
| *Olig2*^12^ | NM_016967.2 | acggcctgctcaagtcac | cccatatattcgactccccata | 346 |
| *Th*^13^ | NM_009377 | caagctcaggaactatgcctct | gagcgcatgcagtagtaagatg | 343 |
| *Trp53*^14^ | NM_011640.3 | tgagccaggagacattttcagg | aagggacaaaagatgacagggg | 237 |
| *Vip*^15^ | NM_011702.2 | tttgaaggagcaggtgaccc | acggcatcagagtgtcgttt | 260 |
| *Vipr1*^16^ | XM_006512068.1 | gaagctgcactgtactcgaaac | gcacaaggatgatgatgatgcc | 418 |
| *Vipr2*^17^ | NM_009511.2 | ctagcgatggatggtcagagac | ccaccagaagccagtagaagtt | 387 |

^1^, Actin beta; ^2^, adenylate cyclase activating polypeptide 1; ^3^, polypeptide receptor 1; ^4^, B cell leukemia/lymphoma 2; ^5^, caspase 3; ^6^, cyclin A2; ^7^, cyclin D1; ^8^, cyclin E1; ^9^, choline acetyltransferase; ^10^, glyceraldehyde-3-phosphate dehydrogenase; ^11^, antigen identified by monoclonal antibody Ki 67; ^12^, oligodendrocyte transcription factor 2; ^13^, tyrosine hydroxylase; ^14^, transformation related protein 53; ^15^, vasoactive intestinal peptide; ^16^, vasoactive intestinal peptide receptor 1; ^17^, vasoactive intestinal peptide receptor 2.
